# Effects of Inoculation with Koji and Strain *Exiguobacterium profundum* FELA1 on the Taste, Flavor, and Bacterial Community of Rapidly Fermented Shrimp Paste

**DOI:** 10.3390/foods13162523

**Published:** 2024-08-13

**Authors:** Huanming Liu, Ailian Huang, Jiawen Yi, Meiyan Luo, Guili Jiang, Jingjing Guan, Shucheng Liu, Chujin Deng, Donghui Luo

**Affiliations:** 1 College of Food Science and Technology, Guangdong Ocean University, Zhanjiang 524088, China; liu241253@gdou.edu.cn (H.L.); 18307631025@163.com (A.H.); 18807647789@163.com (M.L.); lsc771017@163.com (S.L.); chujind@163.com (C.D.); 2 Guangdong Provincial Key Laboratory of Aquatic Products Processing and Safety, Zhanjiang 524088, China; jgl@gdou.edu.cn (G.J.); gjj@gdou.edu.cn (J.G.); 3 College of Food Science and Engineering, Guangdong Ocean University, Yangjiang 529500, China; 18666855764@163.com; 4 Chaozhou Branch of Chemistry and Chemical Engineering Guangdong Laboratory (Hanjiang Laboratory), Chaozhou 521000, China

**Keywords:** shrimp paste, fast fermentation, taste, flavor, correlation, bacterial diversity

## Abstract

This study was conducted to investigate the effect of inoculation with *Exiguobacterium profundum* FELA1 isolated from traditional shrimp paste and koji on the taste, flavor characteristics, and bacterial community of rapidly fermented shrimp paste. E-nose and e-tongue results showed higher levels of alcohols, aldehydes, and ketones, enhanced umami and richness, and reduced bitterness and astringency in samples of shrimp paste inoculated with fermentation (*p* < 0.05). Eighty-two volatile compounds were determined using headspace solid-phase microextraction and gas chromatography–mass spectrometry (HS-SPEM-GC-MS). The contents of 3-methyl-1-butanol, phenylethanol, isovaleraldehyde, and 2-nonanone in the inoculated samples were significantly increased (*p* < 0.05), resulting in pleasant odors such as almond, floral, and fruity. High-throughput sequencing results showed that the addition of koji and FELA1 changed the composition and abundance of bacteria and reduced the abundance of harmful bacteria. Spearman’s correlation coefficient indicated that the alcohols, aldehydes, and ketones of the inoculated fermented samples showed a strong correlation (|ρ| > 0.6) with *Virgibacillus* and *Exiguobacterium*, which contributed to the formation of good flavor in the fast fermented shrimp paste. This study may offer new insights into the production of rapidly fermented shrimp paste with better taste and flavor.

## 1. Introduction

Shrimp paste is a traditional fermented food that is added to dishes as a condiment because of its salty and fresh flavor [1,2]. The traditional method of manufacturing shrimp paste involves adding salt to the mixture and utilizing the microbes and enzymes for spontaneous fermentation [3], during which proteins and fats in the shrimp paste are broken down into small molecules, such as amino acids and fatty acids, which create a delicious and unique flavor [4]. However, the traditional method of producing shrimp paste utilizes high-salt-stress fermentation, which requires a long time to develop a unique flavor, resulting in a long production cycle and susceptibility to factors such as temperature, season, and production personnel, which can affect the quality of the product, especially in terms of flavor [5]. Therefore, shortening the production cycle of shrimp paste and maintaining a good flavor quality are urgent problems in the shrimp paste production industry.

The artificial addition of fermentation agents is commonly used to accelerate the fermentation of a product. Exogenous hydrolytic enzymes, particularly proteases, promote the fermentation of aquatic products [6,7]. Interestingly, koji is the product of solid-state fermentation by *Aspergillus oryzae* (a mold species) using soybeans as the main ingredient. It is rich in enzymes such as proteases, aminopeptidases, and amylases, which are used to break down large molecules, accelerate the fermentation of foods, and create a good flavor. Studies have also shown that the addition of koji improves the flavor, nutrition, and color of fermented products [8]. For example, Kim et al. [9] found that low-salt quick fish sauce prepared with rice koji had better quality, taste, and flavor. However, koji is mostly used in fish sauce and soy sauce and rarely in shrimp paste fermentation. Bacteria are also widely used as fermentation agents for food. The inoculation of the bacteria had a better effect on the fermentation speed as well as the formation of nutrition and flavor of shrimp paste; for example, Prihanto et al. [10] used to ferment terasi with *Lactobacillus plantarum* and *Bacillus amyloliquefaciens* as the starter and demonstrated that the incorporation of both strains improved the physicochemical and sensory characteristics of terasi. To ensure the unique flavor of fermented foods, some researchers have screened microorganisms from fermented foods as starters to improve their flavor (e.g., fish sauce [11], suancai, dry-cured duck [12], and shrimp paste [13]). In addition, our previous work confirmed that *Exiguobacterium profundum* FELA1 screened from traditional shrimp paste could produce proteases and lipases and significantly increase the flavor substances in the enzymatic solution of shrimp heads [14]. Therefore, it can be considered as a fermentation strain for inoculation into shrimp paste to maintain or improve its flavor quality. Rapid fermentation tends to result in a lack of taste and flavor. Therefore, we hypothesized that inoculation with the indigenous *Exiguobacterium profundum* FELA1 and koji might play a role in compensating for the loss of taste and flavor in rapidly fermented shrimp paste.

Flavor is an important indicator of the consumer acceptability of fermented foods. Microorganisms play an essential role in flavor formation in fermented foods. Most current research on shrimp paste focuses on changes in the physicochemical properties, flavor quality, and microflora during the fermentation process of traditional shrimp pastes [15,16]. In recent years, some researchers have begun to focus on the effects of temperature and low salt concentration on changes in bacterial communities during the fermentation of shrimp paste and flavor quality [10,13,17]. However, few studies have analyzed the correlation between bacterial diversity and volatile flavor components in fast-fermented shrimp paste inoculated with fermenters. High-throughput sequencing technology is widely used to detect microbial diversity in fermented foods [18]. The source of microorganisms in shrimp paste relies on the microorganisms in the raw materials and environment, whereas the main microorganisms involved in its fermentation are bacteria [19]. Understanding the correlation between microbiota and flavor is useful for improving the quality of shrimp pastes. Nevertheless, the correlation between bacterial diversity and the flavor quality of shrimp paste microorganisms produced by rapid fermentation with inoculated fermenters is unknown. Therefore, the use of high-throughput sequencing analysis to investigate the correlation between microbial diversity, genus composition, and product flavor is important for the production of rapidly fermented shrimp paste using inoculated fermenters.

In this study, koji and *Exiguobacterium profundum* FELA1 screened from traditional shrimp paste were used to inoculate shrimp paste for rapid fermentation. Traditional shrimp paste was used as the control, and the volatile flavor compounds in the shrimp paste through the two processes were detected and compared using headspace solid-phase microextraction and gas chromatography–mass spectrometry (HS-SPEM-GC-MS). The effects of koji inoculate and screening strains on the taste and volatile components of shrimp paste were also analyzed, and high-throughput sequencing was used to compare the bacterial communities in fast-fermented shrimp paste with those in traditional shrimp paste. The correlation between the dominant bacterial genera and volatile flavor compounds in the two shrimp pastes was preliminarily investigated. This study aimed to contribute to the effective quality control of related products and provides new ideas for the regulation of the production of fast-fermented shrimp paste with better flavor.

## 2. Materials and Methods

### 2.1. Preparation of the Koji

Bran, a soybean meal, was purchased from a regional market. *Aspergillus* oryzae 3.042 culture was purchased from the Guangdong Institute of Microbiology, China. Bran, soybean meal, and water in a conical flask were mixed at a ratio of 4:1:4 (*w*/*w*), sterilized at 121 °C for 30 min, cooled to approximately 30 °C, inoculated with *Aspergillus* oryzae 3.042, and shaken well, sealed with a single layer of gauze at the mouth of the bottle, and then cultured at a constant temperature of 28 °C for 72 h (a tray of water was placed in the incubator to keep the environment moist). The bottles were shaken at regular intervals to keep the agglomerated koji loose until they were covered with mycelium on the surface of the koji after 72 h.

### 2.2. Preparation of Exiguobacterium Profundum FELA1

*Exiguobacterium profundum* FELA1 was isolated from a naturally fermented traditional shrimp head sauce (Zhanjiang, China) and was used as a starter culture. The strain was incubated in a nutrient broth medium (NB, Landbridge, Beijing, China) at 37 °C for 24 h.

### 2.3. Shrimp Paste Preparation

Penaeus vannamei (*Litopenaeus Vannamei*) shrimp heads were obtained from Zhanjiang Guolian Aquatic Products Development Co., Ltd. (Zhanjiang, China), and salt was purchased from a local market.

Traditional shrimp paste (T): The shrimp heads were washed in distilled water, drained of most of the water and then pureed in a blender. Then, 30% (*w*/*w*) salt was added to the crushed shrimp heads, mixed and stirred, and loaded into fermentation tanks. The mixture naturally fermented for 30 days at a temperature of about 30 °C, with stirring every morning and evening during fermentation. After completion of fermentation, samples were collected and stored at −20 °C for GC-MS, e-nose, and e-tongue analysis, and at −80 °C for high-throughput sequencing analysis.

Inoculation of fermentation agent for fermentation of koji shrimp paste (Q): The shrimp heads were washed in distilled water, drained of most of the water, and then pureed in a blender. Then, 30% (*w/w*) salt was added to the crushed shrimp heads, mixed, and stirred into the fermentation bottle. Then, the cultured koji was added, stirred well, covered with a single layer of gauze, and fermented at 36 °C for 48 h. Next, 20% (*w/w*) salt was supplemented, mixed, and inoculated with 6% (*v*/*w*) FELA1 bacteria into the koji shrimp paste, and fermentation was continued at 36 °C for 24 h. Samples were stirred once in the morning and once in the evening during the fermentation stage, and samples were collected after fermentation and stored at −20 °C and −80 °C for follow-up analysis.

### 2.4. E-Nose Measurement

Aromatic compounds in the shrimp paste were detected using an electronic nose (e-nose) (PEN3, Air Sense Analytics GmbH, Schwerin, Germany) according to the method described by Yu et al. [20]. The e-nose consists of 10 sensors, each sensitive to a different odorant. The response characteristics of the odor sensors and their counterparts are listed in Table 1. Specifically, 5 g of the sample was sealed in a 20 mL headspace bottle and allowed to rest for 30 min at room temperature (27 °C). The e-nose probe was then inserted into the bottle to deliver volatile gases to the detector at a constant flow rate of 400 mL/min for 120 s until the sensor signal reached a stable value. To ensure the reliability and stability of the test data, a cleaning procedure was performed for 120 s before testing the sample until the sensor signal returned to baseline. Winmuster 1.6.2 e-nose software was used for data processing and each sample was analyzed in triplicate.

### 2.5. E-Tongue Analysis

The e-tongue test was performed using an SA-402B taste analysis system (Insent, Japan), with some modifications according to the method described by Gao et al. [21]. Five grams of shrimp paste were mixed well with 100 mL of distilled water and then centrifuged at 10,000 rpm for 10 min. The supernatant was diluted with a ratio of 1:1 of supernatant:water and after passing through a 0.45 μm filter membrane, the filtrate was collected. The collected filtrates were placed in special beakers with e-tongues, each with a sample volume of approximately 80 mL. The taste, saltiness, bitterness, bitter aftertaste, astringency, astringency aftertaste, umami, and richness of each sample were detected by the e-tongue at room temperature. The measurement was repeated four times for each sample and three groups of stable data were selected for subsequent analysis.

### 2.6. Measurement of Volatile Flavor Components

#### 2.6.1. Headspace Solid-Phase Microextraction (HS-SPME)

Flavor compounds were extracted from four shrimp paste samples using solid-phase microextraction (SPME), with modifications according to a previously described method [13]. A 5 g sample of shrimp paste was placed in a 20 mL headspace vial and added with 1 μL of the internal standard 2-methyl-3-heptanone (0.8160 mg/mL) and sealed up. It was equilibrated in a stirrer at 60 °C for 10 min, and at the same temperature, the SPME fiber (65 μm PDMS/DVB, Supelco, Bellefonte, PA, USA) was inserted into the headspace vial to absorb the volatiles for 30 min. Then, it was immediately inserted into the GC injection port to be resolved at 250 °C for 5 min.

#### 2.6.2. GC–MS Analysis

The GC-MS analysis was performed using a Shimadzu GCMS-TQ8050NX system (Shimadzu, Kyoto, Japan). Separation was performed on an InertCap Pure-WAX column (30 m × 0.25 mm × 0.25 μm; GL Sciences; Tokyo; Japan). The initial temperature was 40 °C, held for 1 min, raised to 100 °C at a rate of 3 °C/min, held for 5 min, increased to 230 °C at a rate of 5 °C/min, and held for 10 min. Helium was used as the carrier gas at a flow rate of 1.0 mL/min, and the sample was injected without splitting the flow. The EI mass spectra were recorded in the range of 30–480 *m*/*z*.

The NIST17 database was used to compare the detected flavor compounds. When the compound similarity was greater than 80, the content of each compound was calculated based on the ratio of the peak area of the compound to the peak area of the internal standard 2-methyl-3-heptanone. The formula used is as follows:

(1) $$Contents\left(ng/g\right)=\frac{peak\;area\;ratio\times 0.816}{5\left(shrimp\;paste\right)}\times 1000$$

### 2.7. DNA Extraction and Sequencing

Total DNA was extracted from shrimp paste samples using an OMEGA Soil DNA kit (D5625-01; Omega Bio-Tek, Norcross, GA, USA) according to the manufacturer’s instructions. To separate and characterize the extracted DNA fragments, DNA quality was examined using agarose gel electrophoresis, and the resulting DNA concentration was determined using a NanoDrop ND-1000 spectrophotometer (Thermo Fisher Scientific, Waltham, MA, USA). Amplification of the V3-V4 region of the bacterial 16S rRNA gene was performed using universal primers 338F (5′-ACTCCTACGGGAGGCAGCA-3′) and 806R (5′-GGACTACHVGGGTWTCTAAT-3′). PCR and Illumina Miseq sequencing were performed at Shanghai Personal Biotechnology Co., Ltd. (Shanghai, China) according to a previously described method [22].

Sequencing of data was performed using QIIME2 (2019.4). After demultiplexing, the DADA2 plugin was used to filter, denoise, merge, and remove chimeras from sequences. Clustering was then performed with 100% similarity, and de-multiplexed sequences were obtained as amplicon sequence variants (ASVs). The Bayesian classifier with the SILVA Release 132 database was used for taxonomic identification of the processed singleton ASVs. Specifically, mafft [23] was utilized, and a phylogenetic tree was constructed using fasttree2. QIIME2 was used to annotate the screened cross-sectional sequences of each ASV.

### 2.8. Statistical Analysis

Three replicate determinations were performed for the experiments in this study, and the experimental data were statistically analyzed and plotted using Excel and Origin 2021 and PCA and OPLS-DA analyses were performed using SIMCA 14.1 software. Spearman’s rank correlation coefficient was calculated using SPSS, correlation network graphs were visualized by using Cytoscape, and high-throughput data analysis was performed on the Personalbio Genetics Cloud Platform (https://www.genescloud.cn, accessed on 13 December 2023). Differences between the two shrimp paste samples were analyzed using a t-test (*p* < 0.05 was considered to be significantly different).

## 3. Results and Discussion

### 3.1. E-Nose Analysis of Shrimp Paste

**Figure 1:**
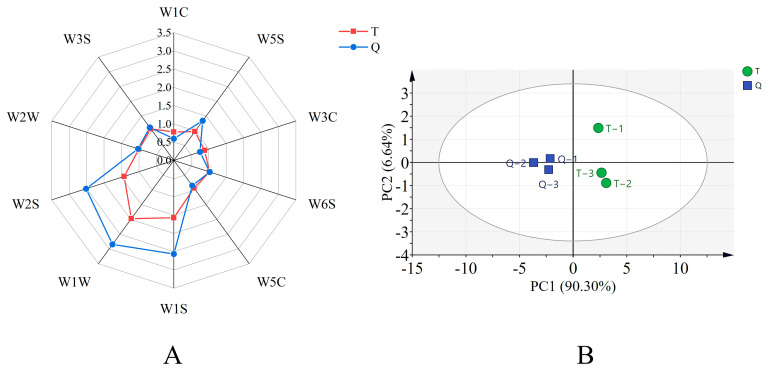
(**A**) Radar diagram of the electronic nose. (**B**) Principal component analysis of electronic data.

The flavors of shrimp paste T and Q were differentiated using an e-nose (Figure 1A,B). This can be observed from Figure 1A and Table 1. All of the sensors responded to the two shrimp pastes; among others, the significant differences (*p* ˂ 0.01) occurred between the response values of shrimp paste T and Q in the W1W, W1S, and W2S sensors, and the latter was significantly higher than the former, indicating that the presence of sulfides, methyl groups, alcohols, aldehydes, and ketones may lead to differences in aromatic properties [24,25]. This result indicated that the e-nose can distinguish between the flavor components of the two shrimp pastes. To gain a deeper understanding of the differences in the composition of volatile components between shrimp pastes Q and T, principal component analysis was performed (Figure 1B). PCA1 and PCA2 accounted for 90.30% and 6.64% of the variation between samples, respectively, with a cumulative contribution of 96.94%, indicating that they could effectively represent the overall information for the samples. PCA was effective in distinguishing the differences between the T and Q values of the shrimp pastes. Briefly, the shrimp paste Q samples were separated from the T samples, indicating that there was a significant difference in flavor between them. Combined with the analysis of the e-nose results, the volatile chemical compositions of the Q and T samples were similar, but the response values of sensors W1W, W1S, and W2S were higher in Q samples, indicating that fermentation of koji shrimp paste by inoculation with strain FELA1 culture increased sulfide, methyl groups, alcohols, aldehydes, and ketones, whereas the response value of sensor W1C was lower than that of shrimp paste T, indicating that it also reduced the production of aromatic compounds and benzenes.

### 3.2. Taste Analysis Based on E-Tongue and Heatmap

**Figure 2:**
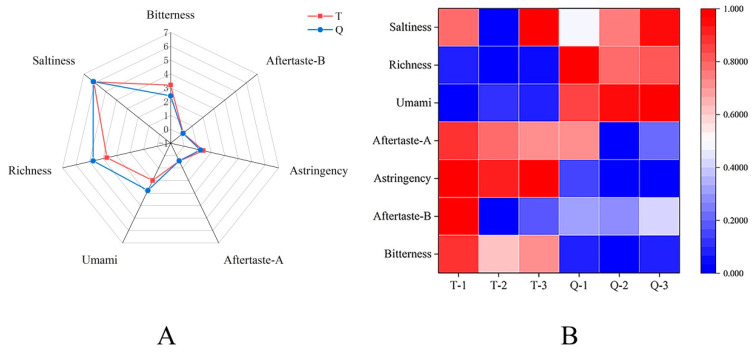
(**A**) Radar diagram analysis of electronic tongues date. (**B**) Heat map analysis of electronic tongue data.

Taste is one of the most important characteristics of fermented foods [26]. It has been shown that tastes such as umami, bitterness, and sweetness are associated with taste-active amino acids and peptides produced by protein hydrolysis or autolysis during food fermentation [27,28]. The e-tongue results and heat map analysis of the shrimp paste samples for both Q and T are shown in Figure 2A,B. The radar map for electronic tongue determination of shrimp paste flavor sensation is shown in Figure 2A. The flavor profiles of shrimp paste Q and T were similar; there were significant differences in bitterness, astringency, umami, and richness (*p* < 0.05), and the response values of both the saltiness and richness sensors were higher than the other sensors, probably because the raw materials are all made up of shrimp heads. The heat map (Figure 2B) showed that the bitterness, astringency, and aftertaste-A were more distinct in shrimp paste T. In contrast, it was found that umami and richness were higher in shrimp paste Q than in T, but bitterness was significantly lower. The above results may be attributed to the action of koji, which contains a rich enzyme spectrum, especially its production of hydrolytic proteases that hydrolyze proteins and produce abundant amino acids, especially umami amino acids (Glutamate) that increase the umami and richness of Q [29,30]. For example, soy sauce uses Aspergillus fermentation to obtain the good savory and umami flavors of the condiment [31]. And aminopeptidase is able to degrade the bitter peptides produced during enzymatic digestion thereby reducing the bitter taste. It has also been shown that the impression of bitterness can be moderated by peptidase activity, as well as by the accumulation of glutamate and related umami-tasting or kokumi-active compounds [27]. Another reason may be that the protein hydrolysis process of the inoculated strains forms various taste substances, such as free amino acids that will increase the abundance of Q [27]. However, the exact reason still needs more in-depth research. Similarly, the umami taste was contrastingly enhanced at higher salinity.

### 3.3. Determination of Volatile Flavor Substances

#### 3.3.1. Measurement of Volatile Components in Q and T

**Figure 3:**
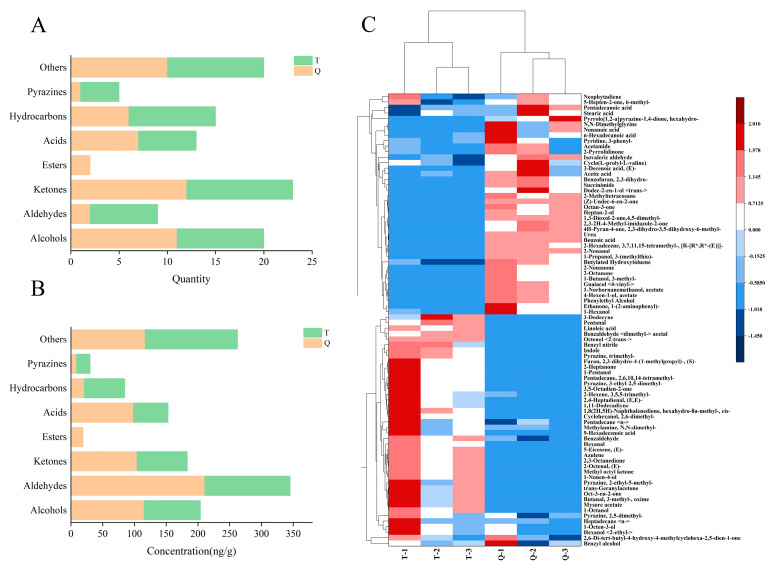
(**A**,**B**) Types and contents of volatile compounds obtained by HS-SPME-GC-MS. (**C**) The content of volatile compounds and clustering results of the 2 shrimp paste samples according to HS-SPME-GC-MS. The color indicates the concentration of the compound, with blue and red indicating low and high concentrations, respectively. The higher the concentration of the compound, the darker the color.

HS-SPME-GC–MS was used to analyze the volatile flavor compounds in the two shrimp paste samples and better understand the rapid fermentation of shrimp paste flavor changes after inoculation. The volatile flavor substances detected in shrimp pastes Q and T and their contents are listed in Table 2. A total of 82 volatile flavor compounds were identified from Q and T. There were 15 alcohols, 7 aldehydes, 19 ketones, 9 acids, 2 esters, 11 hydrocarbons, 4 pyrazines, and 15 other compounds. The quantities of volatile components were similar in both samples (Figure 3A), with 56 volatiles in T and 51 in Q, of which 25 were the same in both samples. However, there were differences in the relative contents (Figure 3B), and inoculation with koji and strain FELA1 for rapid fermentation significantly increased the yield of volatile flavor substances, especially alcohols, aldehydes, and ketones. Furthermore, a heat map cluster analysis was performed to identify the differences between the volatile flavor components of the two shrimp pastes (Figure 3C). The relative contents of the 82 volatile components were processed as variables to obtain a clustered heat map. The frequency of distribution of each substance in the two samples is shown in the comparison along the row.

Alcohols in aquatic products are dominated by polyunsaturated fatty acid (PUFA) degradation [32]. Alcohols were the most extensively detected compounds of the diverse volatile flavor substances in the two shrimp paste samples. In this study, the amount of alcohol detected in shrimp paste Q was higher than in T, but the difference in the number of species was small. We found that 3-methyl-1-butanol, 1-octen-3-ol, phenylethyl alcohol, and hexanol were the major alcohols in shrimp paste Q, whereas the predominant alcohols of T were 1-octen-3-ol, 2,6-dimethylcyclohexanol, 2-ethyl-hexanol, and benzyl alcohol. Additionally, 1-octen-3-ol was detected in both shrimp pastes at high levels. This is also known as mushroom alcohol, has a pleasant mushroom aroma, and is a core flavor alcohol in fermented fish products [11,33,34]. Moreover, the content of 1-octen-3-ol in Q was lower than that of traditional shrimp paste T, which is consistent with the findings of Hiraide et al. [35]. Some studies have shown that this substance is a characteristic flavor substance of ham and mature meat and may be related to the oxidative degradation of fat [33,36]. The substance 3-methyl-1-butanol, which has a fermented aroma, was present at a relatively high level in Q but was not detected in T; it is the main flavor-presenting substance in high-salt diluted soy sauce [37].

Aldehydes usually present a pleasant odor and significantly influence the flavor of shrimp paste products owing to their low odor threshold properties [13,33]. The aldehydes in the two shrimp sauces were less diverse, with benzaldehyde and isovaleric aldehyde as the common substances. Benzaldehyde is obtained by the degradation of branched-chain amino acids under the action of microorganisms and has an almondy, caramelized taste that imparts a pleasant flavor [33,34,38]. In this study, the benzaldehyde content of traditional shrimp paste was higher than that of Q, suggesting that there are microorganisms in the environment that promote the production of benzaldehyde. Pongsetkul et al. [39] recognized it as the major aldehyde in the mature shrimp head paste. Isovaleric aldehydes are associated with the Strecker reaction of amino acids [40] and have a fruity or chocolatey aroma [41]. Moreover, the isovaleric aldehyde content of Q was more than three times that in T. This may be due to the addition of koji during fermentation, which increases its content and thus the aroma. Rapid fermentation also requires some flavor components, such as hexanal, which has a cured meat flavor [42], and some short-chain aldehydes.

The ketones in shrimp paste have a more complicated structure. Ketones are formed by the action of enzymes from microorganisms on lipids or amino acids [19]. In previous studies, they were found to be responsible for the cheese-like flavor in the preparation of fish sauce. Ketones are associated with a higher odor threshold than aldehydes; however, the distinctive floral and fruity flavors they produce affect flavor formation in shrimp paste [43]. In this study, although the ketone species in the bacterially fermented koji shrimp paste Q were lower than those in the traditionally fermented shrimp paste T, they were higher and may have contributed more to the flavor of the shrimp paste. For example, an increase in the content of 2-nonanone, 1-(2-aminophenyl)-ethanone, and 2-octanone in Q provided a pleasant floral and fruity aroma. Meanwhile, pyrazines with lower odor thresholds have a significant influence on the total flavor of fermented foods and are produced mainly through the meladic reaction. Of these 2,5-dimethyl-pyrazine and trimethyl-pyrazine provided shrimp paste with meaty, nutty, and baked aromas similar to baked potatoes. In this study, the pyrazine content in Q was lower than that in T. Rapid fermentation may result in the loss of some aroma components to some extent.

Acid also affects the aroma formation of shrimp paste. The acid content in Q was higher than that in T, and the acetic acid and n-hexadecanoic acid contents were significantly higher in Q samples inoculated for fermentation, probably due to higher temperatures, which is consistent with the findings of Wang et al. [44]. However, with its high threshold value, it contributed little to the aroma of the shrimp paste. Esters have a low threshold; most esters have a fruity and floral aroma, and their contents in the samples were low. However, in the present study, fewer esters were detected in both shrimp sauces, which may be related to the homemade laboratory process and the environment. Hydrocarbons are probably derived from the homolysis of alkoxy radicals in fatty acids, with a smaller contribution to shrimp paste owing to the higher threshold value [20]. In addition, studies have found that the content of fishy substances such as trimethylamine and indole in Q samples was significantly lower than that in T samples, indicating that shrimp paste inoculated with koji and strain FELA1 for rapid fermentation reduced the production of undesirable odors to a certain extent.

Shrimp paste Q had higher amounts of alcohols, aldehydes, and ketones, especially the volatile compounds 3-methyl-1-butanol, 2-nonanone, phenylethyl alcohol, isovaleric aldehyde, and 1-(2-aminophenyl)-ethanone. These chemically important aroma-producing substances are found in a variety of traditional fermented foods that endow the products with floral, fruity, and baking aroma characteristics. Therefore, the inoculated strains for the fermentation of koji shrimp paste not only shortened the fermentation time but also enhanced and improved the flavor of the shrimp paste to a certain extent, and the overall quality was greatly improved despite the loss of certain aroma components.

#### 3.3.2. Analysis of Differences in Volatile Compounds between Q and T

**Figure 4:**
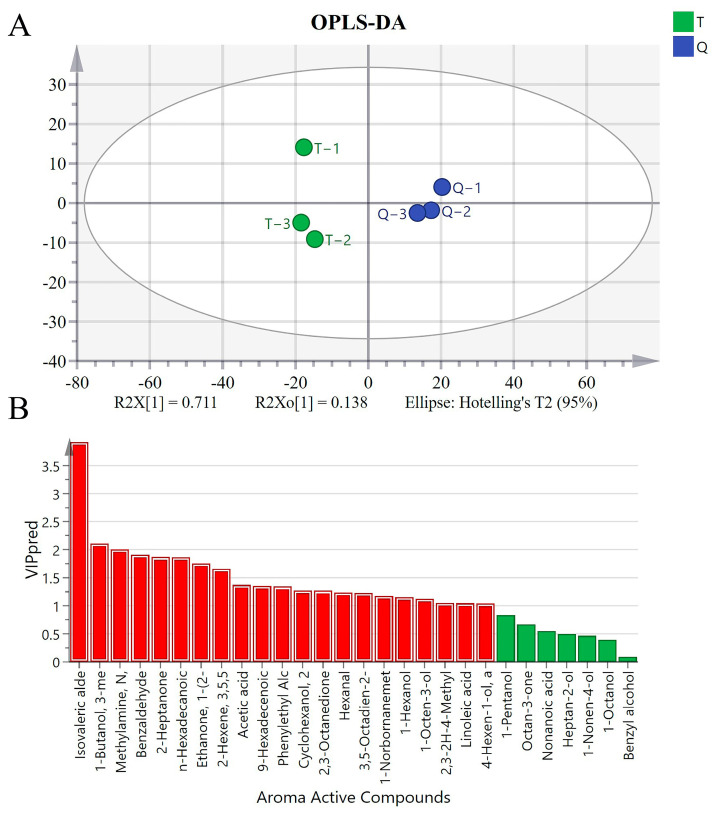
OPLS−DA score (**A**) and VIP scores (**B**) for the two shrimp pastes.

OPLS-DA was used to analyze the differences in odor composition of two shrimp paste types, Q and T. OPLS-DA score plots (Figure 4A, Q2 > 0.95) showed that the six samples were classified into two categories by flavor type, indicating that there were differences in the composition of the odor components of shrimp pastes Q and T. The VIP diagram shows 22 different aromatic substances in the model (Figure 4B, VIP > 1). The results indicate that N,N-dimethyl-methylamine, isovaleric aldehyde, 3-methyl-1-butanol, and 2-nonanone contributed significantly to flavor compositional differences between the two shrimp paste samples.

### 3.4. Analysis of Bacterial Community Diversity

#### 3.4.1. Bacterial Communities of Two Shrimp Paste

A comparison of the bacterial α-diversity indices of the two samples is presented in Table 3. After processing, the total from all samples was 663,260 reads (Including six Q and T samples). After serial quality control processing, 584,110 high-quality serial reads were obtained. Chao1 is the primary indicator of community richness, whereas Shannon and Simpson indices were used to analyze the complexity of species diversity [45,46]. According to these indicators, the diversity index of shrimp paste Q was greater than that of T, suggesting that the microbial community structure of fermented shrimp paste could be changed by the addition of external microorganisms, thereby increasing the diversity of microbial species. The Goods coverage index (GCI) reached 99% (>98%) in both cases, indicating that sequencing information could reveal most of the biological information of the bacterial communities present in both shrimp pastes [47].

**Figure 5:**
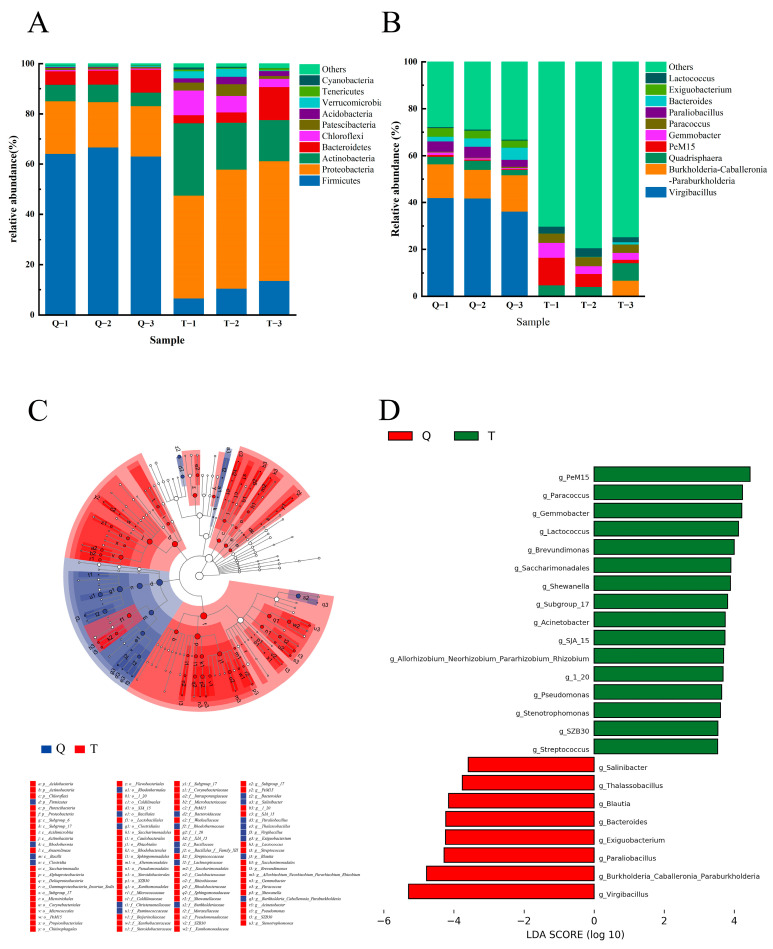
Dominant and differential microorganisms of two shrimp paste species. (**A**, **B**) The relative abundance of microorganisms of phylum and genus in shrimp paste Q and T. (**C, D**) LEfSe analysis of dominant bacteria.

The structure and composition of the microbial communities at the phylum and genus levels in the two shrimp pastes are shown in Figure 5A,B. At the phylum level (Figure 5A), the composition was similar in both shrimp paste samples, with *Proteobacteria*, *Actinobacteria*, *Bacteroidetes*, and *Firmicutes* as the predominant phyla. In addition, these major phyla are also present in other fermented aquatic products such as traditionally fermented fish and fish sauce [18,48]. In Q, the dominant phylum was *Firmicutes* (64.63%). It was also found to be the dominant phylum in traditional Thai fermented shrimp paste [1]. For T, the dominant phylum was *Proteobacteria* (45.34%). *Proteobacteria* contain a large number of pathogens with potential negative impacts on the quality and safety of shrimp paste products. In this study, inoculation of the completely fermented shrimp paste reduced the abundance of *Proteobacteria* and increased the safety of the product compared to traditional shrimp paste.

At the genus level (Figure 5B), *Virgibacillus*, *Burkholderia-Caballeronia-Paraburkholderia*, *Paraliobacillus*, and *Exiguobacterium* were the dominant genera in shrimp paste Q. The dominant genera in traditional shrimp paste T were *Quadrisphaera*, *PeM15*, *Gemmobacter*, and *Paracoccus*. Notably, the abundance of dominant bacterial genera in shrimp paste T was low, whereas the abundance of others was high, probably due to the presence of more miscellaneous bacteria in traditional fermentation methods. After inoculation with koji and strains for rapid fermentation, the bacterial composition (dominant genus) and abundance of shrimp paste Q clearly differed from that of T. This result indicates that different treatments affect the composition of the bacterial community in shrimp paste; for example, Yang et al. [49] found that fermentation at different temperatures had a significant effect on the bacterial community of low-salt shrimp paste, which was manifested in the obvious change of dominant bacterial genera at different fermentation temperatures. Similarly, Huang et al. [50] also found that the composition and abundance of dominant phyla and genera of shrimp paste fermented at different salt concentrations were also changed and also concluded that there was a significant correlation between volatile compounds and bacterial colonization. In the present study, after inoculation with the strains, the abundance of other bacteria in shrimp paste Q was relatively low, while the abundance of *Virgibacillus*, *Exiguobacterium*, etc., increased significantly, which may be due to the competition for nutrients in shrimp paste by the inoculated strains, or the promotion of the growth of salt-tolerant bacteria. Additionally, *Virgibacillus* has good tolerance to higher salt concentrations, and some studies have shown that different *Virgibacillus* can be used as fermentation agents to improve the flavor of shrimp paste [51] or to reduce the histamine content in fish sauce [52].

#### 3.4.2. Differences in the Structure of Q and T Bacterial Communities

Differences in the bacterial flora between the two groups, Q and T, were analyzed using LEfSe (Figure 5C,D). An evolutionary branching map of the differential bacteria using linear discriminant analysis (LDA > 3.5) is shown in Figure 5C. Significant differences in abundance (*p* < 0.05) were identified by mapping the differential bacteria to a taxonomic tree with a known hierarchical structure for 76 species in Q and 23 species in T. Figure 5D indicates that the bacterial genera had significant differences in abundance (*p* < 0.05) in Q and T, suggesting that *Virgibacillus* and *Burkholderia-Caballeronia-Paraburkholderia* could be Q (LDA > 3.5) microbial marker genera. This is in contrast to T (LDA > 3.5), for which *PeM15* and *Paracoccu* are likely to be microbial marker genera.

### 3.5. Correlation between Microflora and Flavor Fompounds

As a result of the differences in bacterial genera and flavor composition of shrimp paste Q and T, Spearman’s correlation coefficient was used to analyze the interactions between dominant bacterial genera (abundance > 1%) and different flavor substances.

A network diagram was constructed based on the correlation coefficients between the 10 microbial bacterial genera and 27 volatile flavor components (|r| > 0.6 and *p* < 0.05)) using Cytoscape for visualization.

**Figure 6:**
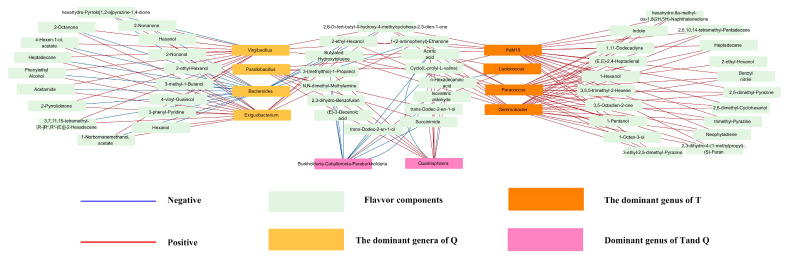
Correlation network between microbial genera and flavor compounds.

The relevance of the bacterial and flavor components is shown in Figure 6. In shrimp paste T, three volatiles were associated with *Lactococcus*, *Burkholderia-Caballeronia-Paraburkholderia*, and *Quadrisphaera*, and *PeM15*, *Gemmobacter*, and *Paracoccus* were positively associated with 23 flavor substances. In shrimp paste Q, a flavor substance was related to *Burkholderia-Caballeronia-Paraburkholderia*, *Paraliobacillus*, and *Quadrisphaera*; 19 odor components were closely related to *Virgibacillus*, *Bacteroides*, and *Exiguobacterium*. Additionally, 3-methyl-1-butanol, 2-nonanone, phenylethyl alcohol, and 1-octen-3-ol were positively correlated with *Virgibacillus* and *Exiguobacterium*. Interestingly, *Bacteroides* were negatively correlated with these substances. Meanwhile, *PeM15*, *Gemmobacter*, and *Paracoccus* were all positively correlated with 1-octen-3-ol in T, which may have resulted in the lower content of 1-octen-3-ol in Q shrimp paste than in T. In addition, studies have found no correlation between the contribution of T and phenylethyl alcohol, whereas most of the dominant genera in Q were positively correlated with this aroma component. Inoculation with strain FELA1 (belonging to *Exiguobacterium*) may have facilitated the production of its contents; therefore, Q has a stronger rose scent than T. Additionally, 3-methyl-1-butanol and 2-nonanone were found only in Q and gave it an aromatic, floral flavor. Despite the positive correlation of the genus with 2-heptanone in T, which also has a floral odor, *Lactococcus* produces a greater fishy odor owing to its positive correlation with N,N-dimethyl-methylamine and its higher content in the samples. The other flavor substances had less impact on the overall aroma of the shrimp paste. Thus, as a whole, the rapid fermentation process of the inoculated strain FELA1 for the fermentation of koji shrimp paste produced similar or better flavors than that of traditional shrimp paste.

## 4. Conclusions

This study demonstrated that inoculation of koji and FELA1 strains for rapid fermentation improved umami and richness and reduced the bitterness and astringency of shrimp paste. The results of the volatile flavor analysis of shrimp paste showed that inoculation with koji and FELA1 did improve the flavor characteristics of fast-fermented shrimp paste. The inoculated shrimp paste sample increased the content of flavor substances (alcohols, aldehydes, and ketones) such as 3-methyl-1-butanol, phenyl ethanol, isovalericaldehyde, 1-(2-aminophenyl)-ethanone, and 2-nonanone with pleasant odors like almond, floral, and fruity odors and decreased the content of fishy and unpleasant odors such as N,N-dimethyl-methylamine and indole (*p* < 0.05). High-throughput sequencing results and the correlation between the dominant bacterial flora and flavor compounds showed that inoculation and fermentation changed the abundance and species of bacteria, reduced the abundance of harmful microorganisms, and increased the abundance of genera that are beneficial for the formation of good flavor qualities in shrimp paste products. *Virgibacillus* and *Exiguobacterium* became the dominant genera after inoculation and were positively correlated with the formation of good flavor substances in the shrimp paste. This research could help provide ideas for inoculating fermenters to modulate the core flora and produce shrimp paste products more quickly. However, the mechanism underlying the rapid fermentation of inoculated koji and FELA1 on the flavor and aroma of shrimp paste and the dominant bacterial genera are not clear, and it is necessary to understand the mechanism of fermentative aroma production and the complex metabolic activities of inoculated koji and FELA1 in future studies.

## Figures and Tables

**Table 1:** Sensor and sensitive substances of the electronic nose.

Sensor	Performance Description	Detection Limits (mg/kg)
W1C	Aromatic hydrocarbon	Toluene, 10
W5S	Nitrogen oxides	NO_2_, 1
W3C	Aromatic ammonia	Benzene, 10
W6S	Hydrogen	H_2_, 0.1
W5C	Alkane aromatic compounds	Propane, 1
W1S	Short-chain alkanes	CH_4_, 100
W1W	Sulfides and terpenes	H_2_S, 1
W2S	Alkane aromatic compounds	CO, 100
W2W	Organic sulfides and aromatic components	H_2_S, 1
W3S	Alkane	CH_3_, 10

**Table 2:** Volatile compounds in two shrimp paste samples.

	CAS	Compounds	Q (ng/g)	T (ng/g)
Alcohols	123-51-3	3-methyl-1-Butanol	35.23 ± 12.22	ND
	3391-86-4	1-Octen-3-ol	21.68 ± 10.78	40.88 ± 20.17
	111-27-3	1-Hexanol	14.15 ± 6.59	2.9 ± 1.25
	60-12-8	Phenylethyl Alcohol	13.99 ± 3.55	ND
	100-51-6	Benzyl alcohol	11.41 ± 2.6	11.15 ± 0.17
	628-99-9	2-Nonanol	6.25 ± 0.48	ND
	505-10-2	3-(methylthio)-1-Propanol	4.67 ± 0.7	ND
	104-76-7	2-ethyl-Hexanol	4.47 ± 1.42	7.7 ± 4.07
	543-49-7	Heptan-2-ol	1.94 ± 0.24	ND
	69064-37-5	trans-Dodec-2-en-1-ol	1.22 ± 0.53	ND
	111-87-5	1-Octanol	ND	1.35 ± 0.67
	18409-17-1	2-trans-Octenol	ND	1.45 ± 0.02
	35192-73-5	1-Nonen-4-ol	ND	1.72 ± 0.42
	5337-72-4	2,6-dimethyl-Cyclohexanol	ND	14.88 ± 7.28
	71-41-0	1-Pentanol	ND	6.95 ± 4.93
Aldehydes	590-86-3	Isovaleric aldehyde	197.14 ± 38.43	61.34 ± 50.09
	100-52-7	Benzaldehyde	13.51 ± 4.77	44.02 ± 6.89
	881395	(E,E)-2,4-Heptadienal	ND	4.3 ± 2.72
	110-62-3	Pentanal	ND	4.31 ± 1.59
	1125-88-8	Benzaldehyde dimethyl acetal	ND	5.6 ± 0.7
	2548-87-0	(E)-2-Octenal	ND	2.44 ± 0.84
	66-25-1	Hexanal	ND	12.73 ± 4.09
Ketones	821-55-6	2-Nonanone	32.01 ± 13	ND
	551-93-9	1-(2-aminophenyl)-Ethanone	31.17 ± 13.84	5.97 ± 2.26
	19179-12-5	hexahydro-Pyrrolo [1,2-a]pyrazine-1,4-dione	9.75 ± 7.45	ND
	39799-77-4	2,3-2H-4-Methyl-imidazole-2-one	9.06 ± 1.33	ND
	616-45-5	2-Pyrrolidinone	4.34 ± 2.62	1.63 ± 0.79
	111-13-7	2-Octanone	4.15 ± 1.46	ND
	106-68-3	Octan-3-one	3.51 ± 0.51	ND
	28564-83-2	2,3-dihydro-3,5-dihydroxy-6-methyl-4H-Pyran-4-one	3.19 ± 0.44	ND
	110-93-0	6-methyl-5-Hepten-2-one	2.38 ± 0.28	1.79 ± 0.74
	107853-70-3	(Z)-Undec-6-en-2-one	1.84 ± 0.18	ND
	37830-90-3	4,5-dimethyl-1,3-Dioxol-2-one	1.68 ± 0.17	ND
	10396-80-2	2,6-Di-tert-butyl-4-hydroxy-4-methylcyclohexa-2,5-dien-1-one	0.77 ± 0.51	0.86 ± 0.39
	110-43-0	2-Heptanone	ND	33.61 ± 21.69
	18402-82-9	Oct-3-en-2-one	ND	1.85 ± 1.03
	3796-70-1	trans-Geranylacetone	ND	1.08 ± 0.62
	38284-27-4	3,5-Octadien-2-one	ND	13.78 ± 6.86
	585-25-1	2,3-Octanedione	ND	13.32 ± 4.73
	693-54-9	Methyl octyl ketone	ND	3.9 ± 1.1
	83406-41-1	hexahydro-8a-methyl-cis-1,8(2H,5H)-Naphthalenedione	ND	1.78 ± 0.87
Acids	21096	n-Hexadecanoic acid	45.51 ± 29.82	9 ± 9.33
	21128	Stearic acid	11.02 ± 2.72	8.01 ± 1.96
	64-19-7	Acetic acid	24.1 ± 12.66	7.04 ± 3.38
	1002-84-2	Pentadecanoic acid	8.11 ± 3.6	3.84 ± 1.58
	65-85-0	Benzoic acid	3.86 ± 0.03	ND
	112-5-0	Nonanoic acid	2.84 ± 1.97	ND
	53678-20-9	(E)-3-Decenoic acid	2.74 ± 2.33	ND
	2091-29-4	9-Hexadecenoic acid	ND	18.41 ± 15.36
	60-33-3	Linoleic acid	ND	8.68 ± 0.93
Esters	91057-8-8	1-Norbornanemethanol, acetate	10.73 ± 3.24	ND
	72237-36-6	4-Hexen-1-ol, acetate	8.48 ± 2.95	ND
Hydrocarbons	26456-76-8	3,5,5-trimethyl-2-Hexene	7.36 ± 3.13	34.81 ± 16.03
	629-62-9	Pentadecane	4.24 ± 0.65	5.77 ± 1.41
	629-78-7	Heptadecane	3.85 ± 0.53	5.83 ± 2.75
	1560-78-7	2-Methyltetracosane	2.5 ± 0.45	ND
	14237-73-1	3,7,11,15-tetramethyl-[R-[R*,R*-(E)]]-2-Hexadecene	1.63 ± 0.23	ND
	504-96-1	Neophytadiene	1.2 ± 0.15	1 ± 0.38
	1921-70-6	2,6,10,14-tetramethyl-Pentadecane	ND	6.92 ± 3.02
	20521-44-2	1,11-Dodecadiyne	ND	0.8 ± 0.44
	275-51-4	Azulene	ND	1.32 ± 0.51
	6790-27-8	3-Dodecyne	ND	4.44 ± 2.36
	74685-30-6	(E)-5-Eicosene	ND	3.14 ± 1.34
Pyrazines	123-32-0	2,5-dimethyl-Pyrazine	8.65 ± 2.85	11.64 ± 2.77
	13360-64-0	2-ethyl-5-methyl-Pyrazine	ND	2.82 ± 1.82
	13360-65-1	3-ethyl-2,5-dimethyl-Pyrazine	ND	2.95 ± 1.62
	14667-55-1	trimethyl-Pyrazine	ND	4.14 ± 1.54
Others	75-50-3	N,N-dimethyl-Methylamine	80.38 ± 4.44	123.66 ± 39.69
	2854-40-2	Cyclo (L-prolyl-L-valine)	11.12 ± 4.31	5.53 ± 3.11
	128-37-0	Butylated Hydroxytoluene	8.36 ± 1.42	3.45 ± 1.51
	1118-68-9	N,N-Dimethylglycine	4.92 ± 3.15	ND
	7786-61-0	4-vinyl-Guaiacol	3.32 ± 0.98	ND
	60-35-5	Acetamide	2.59 ± 1.42	0.9 ± 0.1
	1008-88-4	3-phenyl-Pyridine	2.08 ± 1.33	0.88 ± 0.35
	57-13-6	Urea	1.82 ± 0.01	ND
	123-56-8	Succinimide	1.08 ± 0.42	ND
	496-16-2	2,3-dihydro-Benzofuran	1.02 ± 0.43	ND
	34379-54-9	2,3-dihydro-4-(1-methylpropyl)-,(S)-Furan	ND	2.01 ± 1.25
	120-72-9	Indole	ND	5.52 ± 1.49
	140-29-4	Benzyl nitrile	ND	1.46 ± 0.73
	30772-69-1	Mysore acetate	ND	0.67 ± 0.33
	626-90-4	3-Methylbutyraldehyde Oxime	ND	1.61 ± 0.81

Note: ND means not detected.

**Table 3:** Alpha diversity of two shrimp paste samples.

Sample	Input	Denoised	ASVs Num	Chao1	Shannon	Simpson	Coverage
Q-1	155,369	134,555	1916	1925.83	5.8417	0.88489	0.993311
Q-2	156,763	134,481	1909	1921.31	5.77051	0.881733	0.992922
Q-3	125,327	106,762	1860	1926.3	6.11221	0.905677	0.992477
T-1	76,819	70,188	1813	1839.3	8.70105	0.990417	0.996735
T-2	76,157	70,170	1913	1943.17	8.99986	0.993659	0.9964
T-3	72,825	67,954	732	732.332	7.72582	0.985068	0.999531

## Data Availability

The original contributions presented in the study are included in the article, further inquiries can be directed to the corresponding author.
